# Genetic and Genomic Diversity Studies of *Acacia* Symbionts in Senegal Reveal New Species of *Mesorhizobium* with a Putative Geographical Pattern

**DOI:** 10.1371/journal.pone.0117667

**Published:** 2015-02-06

**Authors:** Fatou Diouf, Diegane Diouf, Agnieszka Klonowska, Antoine Le Queré, Niokhor Bakhoum, Dioumacor Fall, Marc Neyra, Hugues Parrinello, Mayecor Diouf, Ibrahima Ndoye, Lionel Moulin

**Affiliations:** 1 Laboratoire Commun de Microbiologie IRD/ISRA/UCAD, Département de Biologie Végétale, Faculté des Sciences et Techniques, Université Cheikh Anta DIOP de Dakar, Centre de Recherche de Bel Air, Dakar, Senegal; 2 Laboratoire Mixte International Adaptation des Plantes et Microorganismes Associés aux Stress Environnementaux (LAPSE), Dakar, Senegal; 3 IRD-Laboratoire des Symbioses Tropicales et Méditerranéennes (LSTM), Campus de Baillarguet, Montpellier, France; 4 Laboratoire Mixte International Biotechnologie Microbienne et Végétale (LBMV), Rabat, Morocco; 5 Institut Sénégalais de Recherches Agricoles (ISRA), Dakar, Senegal; 6 Irstea, UR MALY, centre de Lyon-Villeurbanne, Villeurbanne, France; 7 MGX-Montpellier GenomiX, Institut de Génomique Fonctionnelle, Montpellier, France; Universidad de Salamanca, SPAIN

## Abstract

*Acacia senegal* (L) Willd. and *Acacia seyal* Del. are highly nitrogen-fixing and moderately salt tolerant species. In this study we focused on the genetic and genomic diversity of *Acacia* mesorhizobia symbionts from diverse origins in Senegal and investigated possible correlations between the genetic diversity of the strains, their soil of origin, and their tolerance to salinity. We first performed a multi-locus sequence analysis on five markers gene fragments on a collection of 47 mesorhizobia strains of *A. senegal* and *A. seyal* from 8 localities. Most of the strains (60%) clustered with the *M. plurifarium* type strain ORS 1032^T^, while the others form four new clades (MSP1 to MSP4). We sequenced and assembled seven draft genomes: four in the *M. plurifarium* clade (ORS3356, ORS3365, STM8773 and ORS1032^T^), one in MSP1 (STM8789), MSP2 (ORS3359) and MSP3 (ORS3324). The average nucleotide identities between these genomes together with the MLSA analysis reveal three new species of *Mesorhizobium*. A great variability of salt tolerance was found among the strains with a lack of correlation between the genetic diversity of mesorhizobia, their salt tolerance and the soils samples characteristics. A putative geographical pattern of *A. senegal* symbionts between the dryland north part and the center of Senegal was found, reflecting adaptations to specific local conditions such as the water regime. However, the presence of salt does not seem to be an important structuring factor of *Mesorhizobium* species.

## Introduction

Soil salinization, which might occur naturally or as a consequence of mismanaged irrigation, is a major and growing environmental problem, especially in arid and semi-arid areas of the world. Approximately 800 million hectares of land worldwide is affected by salt [1]. In Senegal, 6% of lands, mainly in coastal areas are affected by the phenomenon of salinization [2].

Salinization is a desertification factor causing degradation of biological, chemical and physical properties of soils [3]. A consequence of this degradation of soil properties is a decrease in fertility, which leads to a reduction in crop yields, land abandonment, and the loss of natural vegetation replaced by huge expanses of saline areas commonly called “*tanne*” in Senegal. Thus, the supply of services from natural forests becomes insufficient to meet demand, while poverty increases in rural areas.

Reclamation of large areas of saline land through the world seems difficult due to economic and climatic constraints. However, salty soil reclamation could be done using bioremediation method through planting halophyte plants which can absorb salt from soil and enhance the bioproductivity [4,5]. In salted areas of Senegal, despite the disappearance of vegetation islands of legume species with moderate salt tolerance such as *Acacia*, *Prosopis* and *Sesbania* species can be found.

The *Acacia* genus offers several species adapted to degraded environments, particularly in salt affected areas. *A. seyal* Del. and *A. senegal* (L.) Willd. are indigenous woody legumes with important socio-economic roles. First they are widely used in reforestation processes [6] but also for producing gum arabic which is a very important source of income in the Sahel [7,8]. The ability of these species to establish in poor and degraded soils might be due to their aptitude to contract associations with nitrogen-fixing bacteria (rhizobia) and arbuscular mycorrhizal fungi that occur naturally in their rhizosphere [9]. Indeed, symbioses with microorganisms are powerful factors of plant adaptation to adverse environmental conditions, including the lack of major nutrients (nitrogen, phosphorus), biotic (pathogens, phytophagous) and abiotic (drought, salinity) stresses (for review [10]).

Rhizobial populations are known to vary in their tolerance to major environmental factors [11]. It has been reported that salt stress decreases legume growth and nitrogen fixation activity of nodules (for review see [10]). However, inoculation with salt-tolerant strains of rhizobia can enhance the nodulation and nitrogen-fixing ability of the leguminous plants growing under saline conditions, for example in *Acacia* [12–14]. Furthermore, the ability of legume hosts to grow and survive in saline soils was also shown to improve when they were inoculated with salt-tolerant strains of rhizobia [15–17].

Strains of the genus *Mesorhizobium* can establish nitrogen-fixing symbiosis with legume species from temperate, tropical, sub-tropical and arctic areas [18], or associate endophytically with legume plants [19]. Strains isolated from root nodules of the non-legume genus *Parasponia* [20] and different tropical tree legumes, such as *Acacia*, *Leucaena*, *Prosopis*, *Chamaecrista* and *Lotus* in West Africa (Senegal), East Africa (Kenya, Sudan), South America (Mexico, Brazil) and Europe (the Canary island), were described as *Mesorhizobium plurifarium* [21–30]. The large distribution suggests their adaptation to several eco-climatic conditions [31,32]. In a phenotypic comparative study, the *M. plurifarium* type strain was found more tolerant to heat and salt than other type strains of *Mesorhizobium* species [31].

Studies based on the sequencing of the 16S rRNA gene and the 16S–23S intergenic spacer of rhizobial strains associated to *A. seyal* from diverse agro-ecological zones in Senegal reported the predominance of *Mesorhizobium* genomic groups closely related to *Mesorhizobium plurifarium* with putatively several new species that remain to be defined [22,33]. Inferring diversity in the *Mesorhizobium* genus proved to be difficult when using only the 16S rRNA ribosomal taxonomic marker due to its high conservation across *Mesorhizobium* species [34]. The use of alternative phylogenetic markers is thus very important for species definition within this genus, since some species (e.g. *M. mediterraneum* and *M. temperatum* or *M. metallidurans* and *M. gobiense*) cannot be distinguished by their 16S rRNA sequence alone [32,35]. A Multi Locus Sequence Analysis (MLSA) with different core genes has been used previously for phylogenetic purposes in the *Mesorhizobium* genus [35–40].

The average nucleotide identity (ANI) of whole genomes has been recently proposed as an alternative to DNA-DNA hybridizations (DDH) to infer bacterial species affiliation with values of ANI >95% on 69% of conserved DNA matching with the 70% species cut-off of DDH usually kept in taxonomic studies of bacteria [41,42]. The rapid development of bacterial genome sequencing at low cost coupled with comparative genomics software development (using either Blast or Mummer algorithms) as jSpecies [43] or MUMi [44] give opportunities to use such correlations to infer rapidly the species of a given strain.

It is often difficult to correlate the genetic diversity of rhizobia and their tolerance to several stresses as salinity. Several studies have underlined the lack of correlation between the sampling sites characteristics, the genetic diversity of rhizobia and their tolerance to salinity [33,45,46], while others authors could link the origin of the soil with the salt tolerance of rhizobia [47].

In this study we analysed the genetic and genomic diversity of *A. senegal and A. seyal* mesorhizobia symbionts from diverse origins in Senegal and investigated possible correlations between the genetic diversity of the strains, their soil of origin (being under salt-stress or not), and their tolerance to salinity. We first studied at a fine scale the genetic diversity of a collection of mesorhizobia (using Multi Locus Sequence Analysis and genomic fingerprints), inferred their species affiliation using draft genome sequencing and ANI values, and then compared the diversity patterns with salt-tolerance phenotypes and the geographical origin of isolates.

## Materials and Methods

### Bacterial culture and maintenance

**Fig 1:**
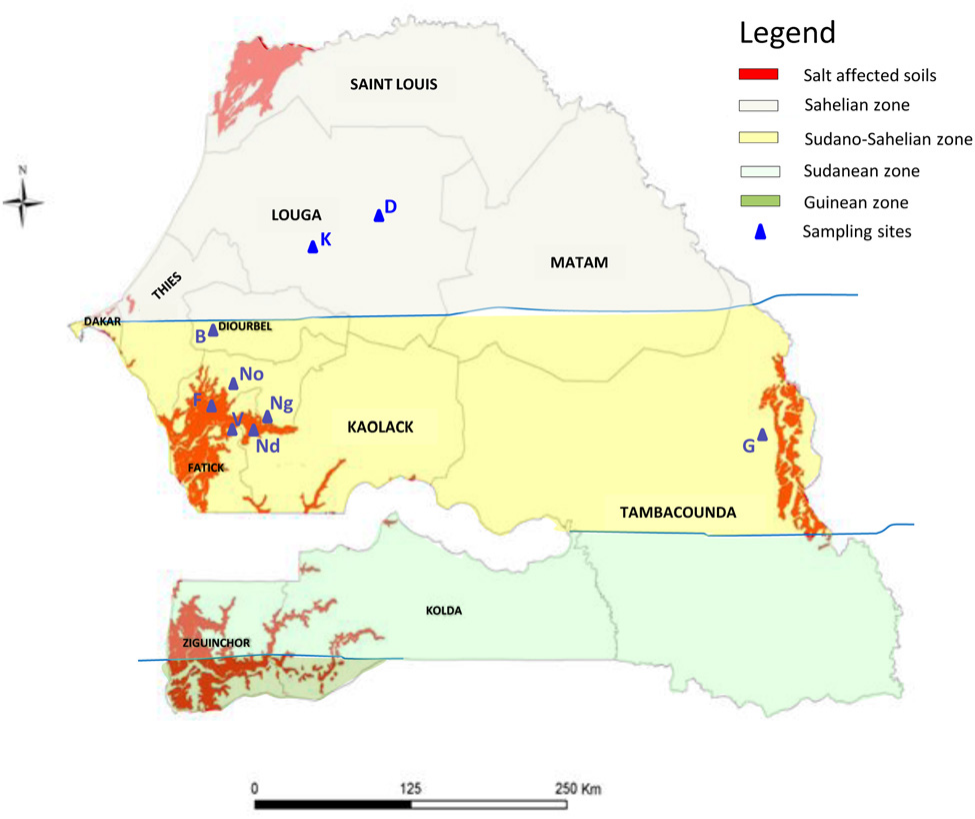
Localization of sampling sites in Senegal. Sampling sites are indicated in blue (D = Dahra, K = Kamb, B = Bambey, F = Foundiougne, Ng = Ngane, V = Vélor, Nd = Ndiafate, No = Nonane, G = Goudiry). The red color indicates salt affected soils (Electroconductivity >4000 μS cm^-1^). Background colors indicate the different climatic zones of Senegal: light yellow = Sahelian zone (annual rainfall 250–500 mm), Dark yellow = Sudano-Sahelian zone (annual rainfall 500–900 mm), light green = Sudanean zone (annual rainfall 900–1100 mm), dark green = Guinean zone (annual rainfall > 1100 mm).

The strains used in this study are listed in Table 1. They originate either from previous studies (See reference in Table 1) or were isolated for this study from nodules on the roots of *Acacia senegal* or *A. seyal* growing in pots on soils collected from the field (rhizospheric soil of *Acacia*). A total of 8 locations (under salt-stress or not, see Fig. 1 and Table 1 for electrical conductivity of soils and gps coordinates), and 36 strains from *A. senegal* and 11 from *A. seyal*, were studied. No specific permissions were required for the sampling locations. All strains were kept in 20% (v/v) glycerol at -80°C and cultured either in TY [48] or YEM [49] media in a shaking incubator at 28°C.

**Table 1:** Bacterial collection of *Acacia* symbionts used in this study.

Species/strain number	Other names	Rep-PCR profile	Tolerance to salt ^£^	Host plant	Geographical origin^$^	Ecogeographical zone	Climatic zone^&^	Soil pH	Soil EC^#^	Référence
Mesorhizobium plurifarium										
**ORS1032^T^**	**LMG11892^T^**	21	400	*A. senegal*	Senegal	ND	Sud	ND	ND	[25]
ORS3302		20	200	*A. seyal*	Ndiafate 2	Groundnut Basin	Sud	6,3	1160	[22]
**ORS3356**		7	300	*A. seyal*	Vélor 2	Groundnut Basin	Sud	6,2	200	[22]
ORS3357		16	200	*A. seyal*	Nonane 1	Groundnut Basin	Sud	5,83	4580*	[22]
**ORS3365**		10	300	*A. seyal*	Foundiougne 5	Groundnut Basin	Sud	6,9	805	[22]
ORS3369		17	300	*A. seyal*	Vélor 1	Groundnut Basin	Sud	6,14	220	[22]
ORS3397		19	300	*A. seyal*	Ndiafate 1	Groundnut Basin	Sud	6,3	1160	[22]
ORS3399		6	300	*A. seyal*	Ngane 1	Groundnut Basin	Sud	4,3	43900*	[22]
ORS3400		ND	300	*A. seyal*	Ngane 1	Groundnut Basin	Sud	4,3	43900*	[22]
ORS3404		8	200	*A. seyal*	Vélor 1	Groundnut Basin	Sud	6,14	220	[22]
ORS3588		18	200	*A. senegal*	Goudiry	Senegal Oriental	Sud	5,96	ND	[23]
ORS3593		3	200	*A. senegal*	Goudiry	Senegal Oriental	Sud	5,96	ND	[23]
ORS3596		9	200	*A. senegal*	Goudiry	Senegal Oriental	Sud	5,96	ND	[23]
ORS3598		ND	200	*A. senegal*	Goudiry	Senegal Oriental	Sud	5,96	ND	[23]
ORS3600		15	200	*A. senegal*	Goudiry	Senegal Oriental	Sud	5,96	ND	[23]
ORS3610		1	200	*A. senegal*	Goudiry	Senegal Oriental	Sud	5,96	ND	[23]
STM8760	K16	14	200	*A. senegal*	Bambey	Groundnut Basin	Sud	6,5	148	This study
STM8770	Dj16	4	300	*A. senegal*	Bambey	Groundnut Basin	Sud	6,5	148	This study
STM8771	Dj17	12	ND	*A. senegal*	Bambey	Groundnut Basin	Sud	6,5	148	This study
**STM8773**	**Dj20**	1	500	*A. senegal*	Bambey	Groundnut Basin	Sud	6,5	148	This study
STM8775	Sd4	6	300	*A. senegal*	Bambey	Groundnut Basin	Sud	6,5	148	This study
STM8777	Sd11	4	400	*A. senegal*	Bambey	Groundnut Basin	Sud	6,5	148	This study
STM8791	Sod14	1	400	*A. senegal*	Bambey	Groundnut Basin	Sud	6,5	148	This study
STM8797	Nd20	4	200	*A. senegal*	Bambey	Groundnut Basin	Sud	6,5	148	This study
STM8799	Da8	2	200	*A. senegal*	Bambey	Groundnut Basin	Sud	6,5	148	This study
STM8805	Ka2	11	200	*A. senegal*	Bambey	Groundnut Basin	Sud	6,5	148	This study
STM8813	Tch9	5	300	*A. senegal*	Bambey	Groundnut Basin	Sud	6,5	148	This study
STM8818	Tch17	5	200	*A. senegal*	Bambey	Groundnut Basin	Sud	6,5	148	This study
*Mesorhizobium* sp. MSP1										
ORS3416		31	200	*A. senegal*	Kamb	Sylvo-pastoral	Sah	5,29	49,4	[24]
ORS3423		32	300	*A. senegal*	Kamb	Sylvo-pastoral	Sah	5,29	49,4	[24]
ORS3437		23	200	*A. senegal*	Kamb	Sylvo-pastoral	Sah	5,29	49,4	[24]
ORS3443		28	200	*A. senegal*	Kamb	Sylvo-pastoral	Sah	5,29	49,4	[24]
ORS3447		26	200	*A. senegal*	Kamb	Sylvo-pastoral	Sah	5,29	49,4	[24]
ORS3448		30	300	*A. senegal*	Kamb	Sylvo-pastoral	Sah	5,29	49,4	[24]
ORS3450		30	300	*A. senegal*	Kamb	Sylvo-pastoral	Sah	5,29	49,4	[24]
ORS3452		22	200	*A. senegal*	Kamb	Sylvo-pastoral	Sah	5,29	49,4	[24]
ORS3573		27	200	*A. senegal*	Dahra	Sylvo-pastoral	Sah	5,97	ND	[23]
ORS3578		25	200	*A. senegal*	Dahra	Sylvo-pastoral	Sah	5,97	ND	[23]
STM8768	Dj14	29	200	*A. senegal*	Bambey	Groundnut Basin	Sud	6,5	148	This study
STM8782	B17	33	300	*A. senegal*	Bambey	Groundnut Basin	Sud	6,5	148	This study
**STM8789^T^**	**Sod10^T^**	28	500	*A. senegal*	Bambey	Groundnut Basin	Sud	6,5	148	This study
STM8792	Sod15	24	400	*A. senegal*	Bambey	Groundnut Basin	Sud	6,5	148	This study
*Mesorhizobium* sp. MSP2										
**ORS3359^T^**	**STM7562^T^**	34	100	*A. seyal*	Nonane 3	Groundnut Basin	Sud	6,29	951	[22]
*Mesorhizobium* sp. MSP3										
**ORS3324^T^**	**STM7563^T^**	35	0	*A. seyal*	Bambey	Groundnut Basin	Sud	6,5	148	[22]
*Mesorhizobium* sp. MSP4										
ORS3428		37	200	*A. senegal*	Kamb	Sylvo-pastoral	Sah	5,29	49,4	[24]
ORS3628		36	400	*A. senegal*	Dahra	Sylvo-pastoral	Sah	5,97	ND	[23]

Strains in bold indicate strain which genome was sequenced in draft. STM, culture collection of Laboratoire des Symbioses Tropicales et Méditerranéennes Montpellier, France. ORS, culture collection of the Laboratoire Commun de Microbiologie IRD/ISRA/UCAD Dakar, Senegal.

£: Tolerance to salt in mM as estimated in this study: the number indicates the concentration of salt tested at which the strain still grows.

&: Sud: Sudano-sahelian zone with 500–900 mm of annual rainfall; Sah: Sahelian zone with 300–500 mm of annual rainfall.

#: Soil ElectroConductivity (EC) in μS cm^-1^.

* indicated salted soil according to FAO (>4000 μS cm-1.).

$: Site with numbers corresponds to sites in [22].

GPS coordinates: Ndiafate 1: 14°03 753’N, 16°11 239’W; Ndiafate2: 14°03 914’N, 16°11 200’W; Velor1: 14°03 467’N, 16°11 243’W; Velor2: 14°03 466’N, 16°11 176’W; Nonane1: 14°18 187’N, 16°21 340’W; Nonane3: 14° 18 144′N, 16°21 163’W; Foundiougne5: 14°11 935’N, 16°26 836’W; Ngane1: 14°35 N, 16°42 W), Goudiry: 14°11N, 12°43W, Bambey: 14 42’N, 16 28’W; Kamb: 15 31′N, 15 25′W; Dahra: 15°21N, 15°29W. See [23–24] for detailed description of sampling sites.

### Phenotypic tests

Tolerance to sodium chloride (NaCl) of rhizobia was tested in 96 well microplates (Nunc Microwell) in broth TY medium [48]. Microplates containing medium supplemented with increasing amounts of NaCl (0 to 600 mM) were inoculated with pure rhizobial culture suspensions (inoculation with 10 μl at optical density (OD) of 1 in a final volume of 200 μl per well, to reach OD 0.05) and incubated on a rotary shaker (160 rpm) at 28°C. Growth was monitored during 72 h by measuring the OD at 600 nm using a microplate spectrophotometer (TECAN-Infinite M200).

### Molecular methods

**DNA extraction, PCR and sequencing**. DNA extraction was performed using a K proteinase lysis protocol as previously described [50]. All PCR amplifications were performed with Go-Taq polymerase (Promega) following manufacturer instructions. The primers used for PCR and sequencing are described in Table 2. The 16S ribosomal DNA (rDNA) was amplified using the universal eubacterial primers FGPS6 and FGPS1509 [51]. The 16S rDNA amplification was carried out as previously described [52]. Fragments of the house-keeping genes *atp*D, *dna*J, *gyr*B, and *rec*A were amplified as described before [35,39]. The *gln*A gene was amplified using either GSI3–58F and GSI2–1143R [53] primers or *glnA572F* and *glnA1143R* [54]. PCR products were purified and sequenced by Genoscreen Inc.

**Table 2:** Primers used in this study for PCR and Sanger sequencing.

Targets	Primers	5’-3’ sequences	Product (bp)	Reference
16S rDNA	FSGP6	GGAGAGTTAGATCTTGGCTCAG	1500	[51]
	FGPS1509	AAGGAGGGGATCCAGCCGCA		
*atpD*	atpD-F	ATCGGCGAGCCGGTCGACGA	470	[35]
	atpD-R	GCCGACACTTCCGAACCNGCCTG		
*dnaJ*	dnaK-F	CAGATCGAGGTSACCTTCGAC	1600	[39]
	dnaJ-R	CGTCRYCATMGAGATCGGCAC		
*glnA*	glnA572F	GGACATGCGYTCYGARATGC	530	[54]
	glnA1142R	TGGAKCTTGTTCTTGATGCCG		
	GSI3–58F	GAYCTGCGYTTYACCGACC	1085	[53]
	GSI2–1143R	GTCGAGACCGGCCATCAGCA		
*gyrB*	gyrB-fornew	TGCTGCTCACCTTCTTCTTCCG	695	[39]
	gyrB-revnew	CCYTTGTAGCGCTGCATGGT		
*recA*	recA-F	ATCGAGCGGTCGTTCGGCAAGGG	440	[35]
	recA-R	TTGCGCAGCGCCTGGCTCAT		
Rep PCR	Rep1R-1	IIIICGICGICATCIGGC	200–5000	[56]
	Rep2–1	ICGICTTATCIGGCCTAC		

**Draft genome sequencing and assembly**. For whole genome sequencing, strains were grown in 50 ml of broth Yeast-mannitol medium and DNA isolation was performed using a CTAB (Cetyl trimethyl ammonium bromide) bacterial genomic DNA isolation method. DNA quality and quantity was evaluated on a Nanodrop spectrophotometer. The draft genome of 7 strains was produced using Illumina Hiseq technology at Montpellier Genomix platform (MGX), with paired-end sequencing on 700 bp fragments, read length of 100 bp, at 2000X coverage (1/3 Illumina lane per bacteria, around 60 million reads per strain). Sequences were assembled on CLC Genomic workbench v5, and contigs were filtered on size (>500 bp) and reads coverage (minimum of 200X, average cover at 2000X). Genomes were automatically annotated using the Microscope platform [55]. The comparative genomic study of the 7 strains is part of a separate article.

**Rep PCR amplification**. Repetitive extragenic palindromic PCR (Rep-PCR) genomic fingerprinting was generated with primers REP1R and REP21, as previously reported [56]. PCR mix and conditions were as described by Mishra et al [57]. Rep PCR amplification product were electrophoresed in a 1% agarose gel in TAE 1X buffer and 0.5 μg of ethidium bromide per ml and photographed under UV light.

### Phylogenetic analyses

Gene fragments sequences were corrected with Chromas Pro v1.33 software (Technelysium) and aligned using either ClustalX [58] or muscle as implemented in MEGA6 [59]. Alignments were corrected manually under Genedoc software [60] when necessary, and recombination in the datasets was evaluated using the Recombination Detection Program (RDP) v4.35 [61]. Recombination was inferred as true when at least two programs of RDP (RDP, Geneconv, Bootscan, MaxChi, Chimaera, Siscan or 3Seq) could detect the same event. Single marker phylogenies were built with MEGA6 using either Neighbor-Joining (with Kimura 2 distance correction method [62] or Maximum likelihood analyses with 1000 bootstrap replicates. A Bayesian phylogenetic tree was built from the concatenate of all 5 gene fragment alignments, using a Markov chain Monte-Carlo (MCMC) analysis. The priors used for the MCMC analysis were based on a GTR+I+G model with 6 types of substitutions, with parameters estimated by maximum likelihood with Modeltest 3.6 [63].

### Average nucleotide identities of whole genomes

Whole genome comparisons using Average Nucleotide Identities (ANI) between genome sequences were produced using jSpecies [43] that use both Blast and Mummer alignments to evaluate whole genome homologies. A blast approach on 1000 bp windows was preferred due to the draft status of genomes. Available genomes of *Mesorhizobium* were included in the analysis (see Table 3 for Accession numbers). Cut-offs for species delineation were 95% ANI on 69% of conserved DNA according to Goris *et al*. [41].

**Table 3:** Genome characteristics of strains sequenced as part of this study as well as reference genomes used for average nucleotide identities calculations (ANI).

Bacterial strain	Other names	GOLD id^£^ / Genbank	Project AN in EBI^$^/Study ID or Genbank AN	NCBI Taxon ID^§^	Contig Count	Estimated Size (in Kb)	Gene Count
**Sequenced in this study**							
*M. plurifarium* ORS1032^T^		Gb0028550	PRJEB6720/ERS517733	69974	52	7108	7293
*M. plurifarium* STM 8773	DJ20	Gb0091368	PRJEB6723/ERP006361	69974	46	7144	7425
*M. plurifarium* ORS 3356	STM 8724	Gb0091369	PRJEB6721/ERP006359	408182	76	7646	8097
*M. plurifarium* ORS 3365	STM 8727	Gb0091371	PRJEB6722/ERP006360	408185	67	7240	7618
MSP1 STM 8789	Sod10	Gb0091370	PRJEB6724/ERP006362	68287	61	6515	6766
MSP2 ORS 3359	STM 7563	Gb0091372	PRJEB6726/ERP006364	408184	214	6885	7191
MSP3 ORS 3324	STM 7562	Gb0091373	PRJEB6725/ERP006363	408180	242	6585	7012
**Reference genomes used for ANI**							
*M. loti* USDA3471^T^	NZP2213	Gb0010058	NZ_AXAE00000000.1	935547	3	6529	6375
*M. huakuii* MAFF303099^%^		Gb0000748	NC_002678.2	266835	3	7596	7334
*M. opportunistum* WSM2075^T^	LMG 24607	Gb0003955	NC_015675.1	536019	89	6884	6747
*M. ciceri* WSM1271		Gb0003892	NC_014923.1	765698	2	6690	6532
*M. australicum* WSM2073^T^	LMG 24608	Gb0003957	NC_019973.1	754035	19	6200	6076
*M. amorphae* CCNWGS0123		Gb0014286	NZ_AGSN00000000.1	1082933	274	7293	7136
*M. alhagi* CCNWXJ12–2^T^		Gb0020563	NZ_AHAM00000000.1	1107882	375	6968	7244

^£^: Gold id refers to the Genome on-line database (http://www.genomesonline.org/);

^$^: Project id at EBI (http://www.ebi.ac.uk/); genomes were submitted to the European Nucleotide Archive database under this project ID.

§: NCBI taxon number at http://www.ncbi.nlm.nih.gov/taxonomy.

%: Strain MAFF303099 was assigned to the *M. huakuii* species in previous reports [65,66]. AN: Accession Number.

### Statistical analysis

Basic statistics as well as multiple correspondence analyses (MCA) were conducted in R v3.1.0 software using Ade4 and FactoMineR modules. Quantitative variables (soil pH, soil electro-conductivity and strain tolerance to salinity were transformed into qualitative variables (using different classes). We tested for significant differentiation of populations (in terms of species proportion) between the two sahelian and sudanian climatic zones, and among the three groups of salt tolerance that contained at least three individuals (200, 300 and 400 mM). Unbiased P values were estimated with an exact G tests implemented in Genepop [64].

### Accession numbers

The sequences determined in this study have been deposited in the GenBank database and accession numbers are indicated in S1 Table: 16S rRNA (KJ609577-KJ609603); *atp*D (KJ648182-KJ648227); *dna*J (KJ648228-KJ648270); *gln*A (KJ883298-KJ883340); *gyr*B (KJ648271-KJ648315); *rec*A *(*KJ609604-KJ609649). Genome assemblies produced in this study have been deposited in the European Nucleotide Archive under Project numbers PRJEB6721 to PRJEB726 and accession numbers ERP006359 to ERP006364.

## Results

### Multi-Locus Sequence Analysis of the *Mesorhizobium* collection isolated from two *Acacia* species from salt-contrasted soils

**Fig 2:**
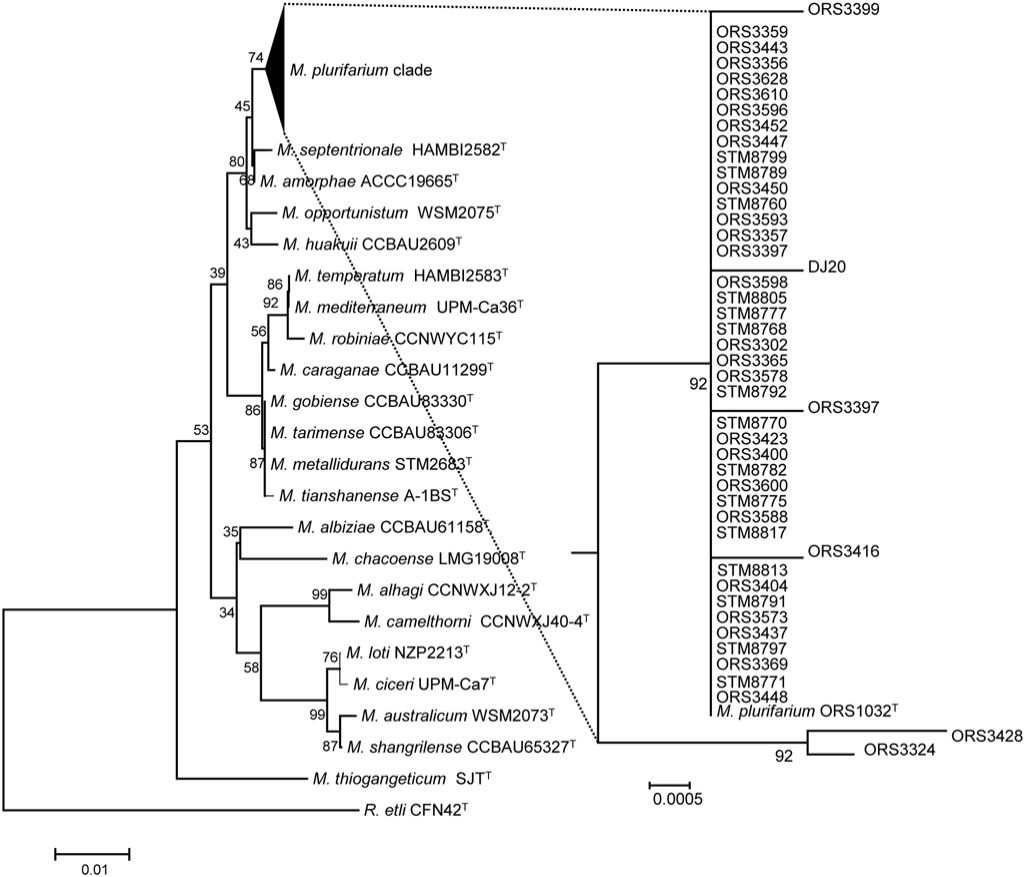
Phylogeny of the 16S rRNA marker of the *Mesorhizobium* collection, built by neighbor joining from a distance matrix corrected by the Kimura-2 method. The scale bar indicates the number of substitutions per site. Numbers at tree nodes indicate % of bootstrap replicates.

We first sequenced 1000 bp of the 16S rRNA marker (encompassing its variable part) in all strains (except those already sequenced) and produced a 16S rRNA phylogeny presented in Fig. 2. The 16S-derived phylogeny was not well resolved within the *Mesorhizobium plurifarium* clade, with poor bootstrap values at mostly all branch nodes, and long branches for several strains indicating nucleotide divergence.

In order to better resolve the genetic diversity of the collection, we produced a multi locus sequence analysis on five house-keeping gene fragments (*rec*A, *gyr*B, *gln*A, *dna*J, *atp*D). Gene markers were chosen according to their use and performance in previous *Mesorhizobium* diversity studies. The vast majority of gene fragments could be amplified and sequenced in almost all strains using methods and primer sets listed in Mat&Methods and Table 2, respectively. Aligned partitions of each gene were built, including reference strains (either type strains of *Mesorhizobium* species and available genomes). The list of reference strains used is presented in S1 Table. Recombination within datasets was assessed to remove sequences displaying horizontal gene transfer (see Mat&Methods for details). Recombination was detected in *gyr*B of ORS3369, *gln*A of ORS3404, and *dna*J of ORS3448. These sequences were removed from the datasets to avoid conflicting phylogenetic signals.

**Fig 3:**
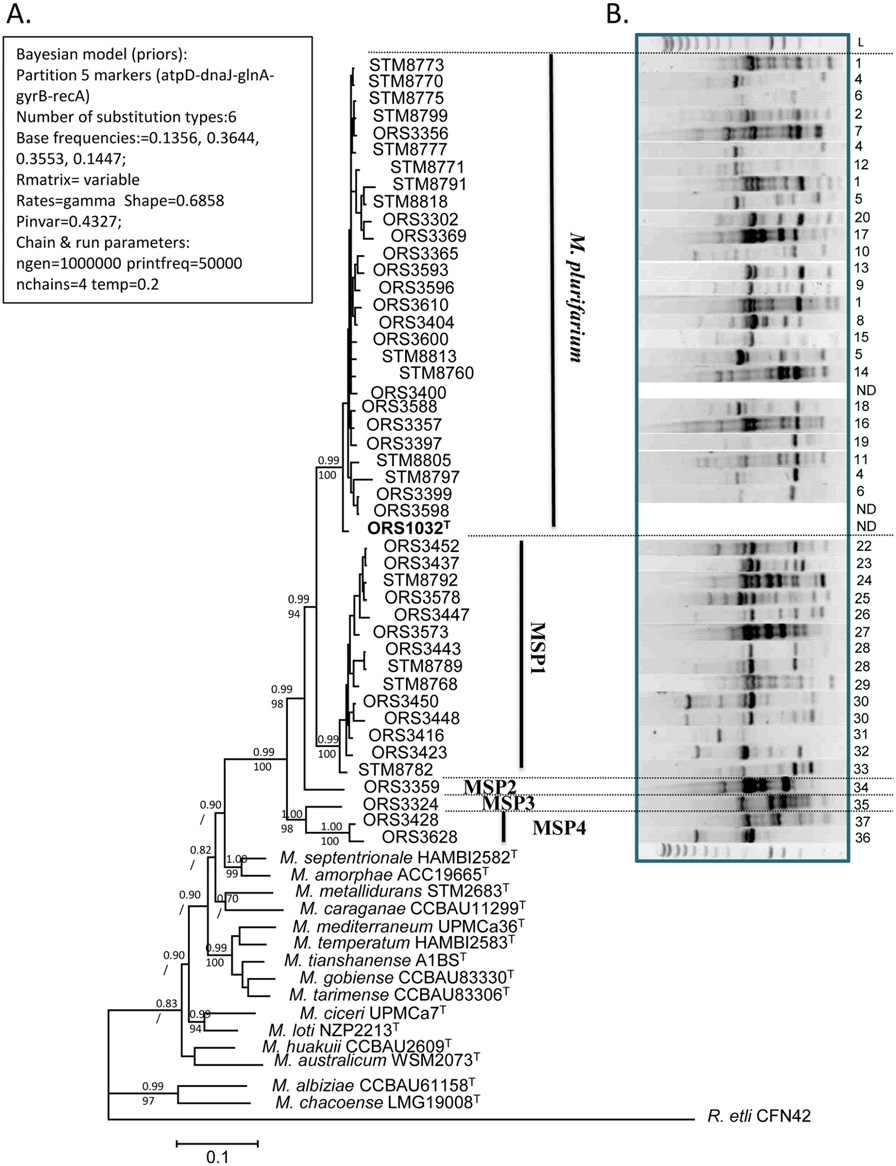
Bayesian phylogeny of the 5 markers alignment (*atp*D-*dna*J-*gln*A-*gyr*B-*rec*A) (A) and rep-PCR fingerprints (B) of the *Mesorhizobium* collection. The priors (estimated by ML) and run parameters for the Bayesian phylogeny are indicated on the left side of the tree. The tree shown is a 50% majrule consensus of all trees produced by the 4 Markov chains. Posterior probabilities (upper number) and bootstraps from 1000 replicates from a ML phylogeny (below number) are indicated at each tree nodes (/ indicate that there is no bootstrap available because the tree node was not common between the Bayesian tree and the ML tree). The scale bar indicates the number of substitutions per site. For the rep-PCR fingerprints (B), see the Mat&Methods section. Each profile was numbered on the right side of the gel. Rep-PCR profiles were not obtained for ORS3400, ORS3598 and ORS1032.

The alignments of all genes were concatenated to produce a global alignment of 2637 bp, with *atp*D (1–421 bp), *dna*J (422–1195 bp), *gln*A (1196–1606 bp), *gyr*B (1607–2237 bp) and *rec*A (2238–2637 bp). A Bayesian phylogeny was then built using the prior (estimated by ML) and run parameters as shown on Fig. 3A, and a consensus tree was built. Bootstraps from 1000 replicates obtained from another analysis by maximum likelihood (the ML model being the same than used for the priors of the Bayesian study on all markers) were added to the tree nodes on Fig. 3A. The phylogenetic tree obtained was much more resolved than the 16S rRNA tree, with *Mesorhizobium* strains from *A. senegal* and *A. seyal* being splitted in five clades supported by high posterior probabilities and bootstraps from the Bayesian and ML analyses, respectively. These clades were named *M. plurifarium* (MP) for clade I, and MSP1 to MSP4 for the others clades as they did not include any known type strain of *Mesorhizobium* species. The first clade includes the highest number of strains (29) from the collection together with the type strain of *Mesorhizobium plurifarium* ORS 1032^T^. This clade includes both strains isolated from *A. seyal* (9 strains) and *A. senegal* (20 strains), unlike other clades which contain only strains isolated from *A. senegal* (MSP1, MSP4) or *A. seyal* (MSP2, MSP3). The clade MSP1 includes 14 strains, while MSP2 to MSP4 were rare as they comprise one to two strains.

### Genomic diversity of bacteria assessed by Rep-PCR fingerprints

Molecular typing of *Mesorhizobium* strains by Rep-PCR amplification generated multiple amplification products ranging in size from 200 bp to 5000 bp (Fig. 3B). The results show a high intraspecific variability in the *Mesorhizobium* clades, with very few identical genome fingerprints among strains. This result mirrors the high number of sampling sites and species diversity and the almost absence of clonal isolates in our collection.

### Genome sequencing of representative strains & Average Nucleotide identities

Draft genomes of strains were produced in order to evaluate the genomic diversity of *M. plurifarium* strains nodulating *A. seyal* and *A. senegal* as well as to determine if several species were present in the collection. Whole genome of four strains of *M. plurifarium* (including the type strain ORS1032^T^) and three strains of *Mesorhizobium* sp. belonging to clade MSP1 to MSP3 were sequenced. No genome was chosen in clade MSP4 as our preliminary analyses did not detect this new clade, and our criteria included also the salt tolerance and strains in this group did not exhibit interesting phenotypes. Information on the sequencing method is given in the Mat&Method section, and descriptive information on the genomes can be found in Table 3.

Average nucleotide identities (ANI) were calculated to evaluate species affiliation of strains which genomes were sequenced. According to Richter and Rossello-Mora [43] there is a correlation between the percentage of average nucleotide identity (ANI %) and the percentage of DNA-DNA hybridization, which is a major criteria in bacterial species delineation. The Table 4 contain the matrix of nucleotide identities between whole genomes calculated under jSpecies [43] by the blast method on windows of 1000 bp in size. We colored in gray the ANI% value of strains which genomes identities exhibit the criteria of belonging to the same species as defined by Goris *et al* [41]. The latter article reported that when strains share an ANI % > 95% on more than 69% of conserved DNA, they would belong to the same species. According to these criteria, ORS3356, ORS3365 and DJ20 share more than 95% ANI with the type strain of *M. plurifarium* ORS1032^T^, and are classified in clade MP in the MLSA tree. The strains STM8789, ORS3359 and ORS3324, respectively belonging to MSP1, MSP2 and MSP3, did not share these criteria with any *Mesorhizobium* species included in the analysis.

**Table 4:** Average nucleotide identities (ANI) between *Mesorhizobium* genomes

	ORS1032	ORS3356	ORS3365	Dj20	Sod10	ORS3359	ORS3324	USDA3471	MAFF303099	WSM2075	WSM1271	WSM2073
*M. plurifarium* ORS1032^T^	---	**96.01 (78.86)**	**96.0 (78.11)**	**95.97 (77.89)**	90.84	89.64	86.01	81.88	82.04	81.85	81.44	81.65
*M. plurifarium* ORS3356	**95.81 (74.04)**	---	**97.18 (83.86)**	**96.62 (80.98)**	90.85	89.83	86.85	81.71	81.81	81.74	81.06	81.41
*M. plurifarium* ORS3365	**96.12 (76.53)**	**97.54 (87.41)**	---	**97.03 (84.13)**	91.28	89.95	86.49	81.9	82.02	81.93	81.26	81.56
*M. plurifarium* Dj20	**96.06 (77.53)**	**97.0 (85.35)**	**97.07 (85.05)**	---	91.9	89.66	86.21	81.94	81.97	81.94	81.26	81.54
MSP1 Sod10	91.24	91.43	91.5	92.03	---	89.48	86.44	81.83	81.96	82.0	81.39	81.65
MSP2 ORS3359	89.99	90.44	90.25	90.0	89.53	---	86.96	82.11	82.26	82.05	81.39	81.59
MSP3 ORS3324	86.43	87.33	86.78	86.48	86.52	87.01	---	82.23	82.39	82.08	81.74	81.77
*M. loti* USDA 3471^T^	82.16	82.02	82.04	82.1	81.85	81.89	82.07	---	89.26	87.14	85.35	86.11
*M. huakuii* MAFF303099	82.42	82.14	82.21	82.23	81.9	82.3	82.37	89.35	---	87.74	85.76	86.43
*M. opportunistum* WSM2075	82.21	82.2	82.18	82.21	82.08	82.09	82.04	87.37	87.83	---	87.97	88.11
*M. ciceri* WSM1271	81.82	81.53	81.58	81.55	81.3	81.38	81.79	85.49	85.81	87.87	---	86.72
*M. australicum* WSM2073	82.23	81.97	81.9	82.0	81.77	81.71	81.9	86.46	86.76	88.25	86.99	---
*M. amorphae* CCNWGS0123	82.93	82.84	82.71	82.91	82.72	82.76	83.28	83.61	84.03	83.81	83.64	83.16
*M. alhagi* CCNWXJ12–2	76.8	76.55	76.55	76.64	76.4	76.31	76.58	76.47	76.61	76.52	76.47	76.41

% of ANI were calculated using blastn on 1000 bp windows of the genomes, with jSpecies (see Math&Methods). In bold are indicated ANI matching with the species affiliation cut-off: >95%, with the % of conserved DNA indicated between parentheses (>69%), as defined by Goris et al. and Konstantinidis & Tiedje [41,42].

### Tolerance to salinity of the collection

**Fig 4:**
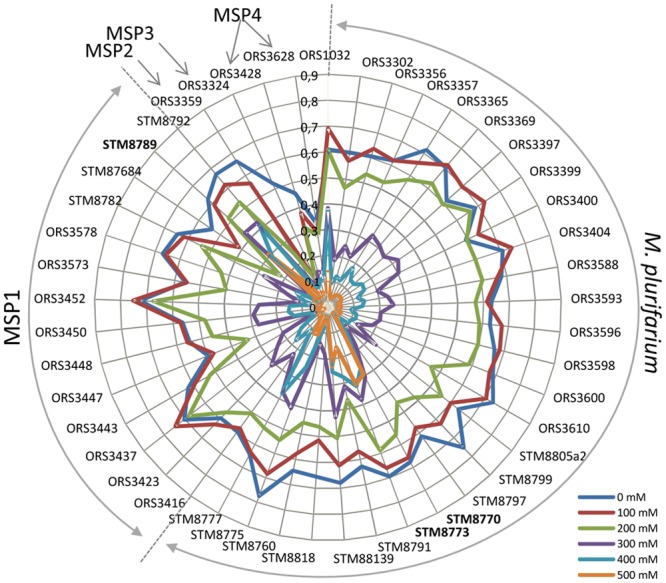
Radar of *Mesorhizobium* strain optical density when grown in TY broth medium in 200 ul in a 96 well microplate with different concentrations of NaCl (100 to 500 mM). The data presented here are at 41h post-inoculation with 0.05 OD as a starting point. Radars at 24h and 68h are presented in S1 Fig., and histograms of growth for each strain at 41h are presented in S2 Fig.

Salt tolerance test of all strains (in triplicates) were conducted in microplate in TY broth medium supplemented with 0 to 600 mM of NaCl. Our aim was to establish putative correlations between the salt tolerance phenotype, the species affiliation and/or geographical origin of the isolates. We considered that tolerance of a given strain to a particular concentration of salt was true when the bacteria could still grow at 50% compared to its growth in the same medium without salt (considered as the 100% of growth). As the collection was quite high in number with tests in 7 different concentrations (0, 100, 200, 300, 400, 500 mM) and 3 OD sampling times (24, 41 and 68 hours), we chose to represent the data of the OD growth of the strain at 41h post inoculation of the medium, using a radar graphic, shown in Fig. 4. The same radar at 24h and 68h is presented in S1 Fig., and histograms of growth% compared to the unsalted control with standard deviation are given for every strain in S2 Fig. For *M. plurifarium* (MP) and MSP1 species, half of the strains were highly impacted in their growth at 300 mM (growth <50% compared to control) and most could not grow (OD not exceeding 0.3) at 400 mM of salt in the medium. Some strains exhibited a better tolerance to salinity and could still grow up to 400 mM (ORS1032, Dj20, Sd11, Sod14, Sod10 and Sod15). Some strains could even grow at 500 mM of salt: Dj16 and Dj20 for MP, Sod10 for MSP1. The few strains of MSP2, MSP3 and MSP4 were poorly tolerant to salt (even at 200 mM), and strains of MSP4 did not even grew well at low salt concentrations, probably due to the growth conditions that might be unadapted to this species.

### Correspondence Analyses between salt responses, genetic typing and the origin of strains

We performed a multivariate correspondence analysis (MCA) in order to study the distribution of our strains according to the following parameters: tolerance of strains, species, plant host, and pedoclimatic conditions of the sampling sites (soil pH and electro-conductivity (EC), geographical origin, eco-geographical and climatic zones). Quantitative variables were transformed in qualitative variable using different classes with biological relevance (pH, tolerance to salinity, EC). The results are presented as a MCA plot in S3 Fig. (A) and factors from the MCA are represented individually in S3 Fig. (B). As observed, there was no correlation between the distribution of strains and their salt tolerance, their species affiliation, and/or their geographical or soil of origin (being salted or not). We tested for significant differentiation of populations (in terms of species proportion) between the two sSahelian and Sudano-sahelian climatic zones, and among the three groups of salt tolerance that contained at least three individuals (200, 300 and 400 mM). There was no significant difference among the three groups of salt tolerance (all pairs of population, p values from 0.45 to 1). Conversely, the two climatic zones (marked by different annual rainfall amounts) were significantly differentiated (p = 0), reflecting a different composition of species between them. Indeed the MP, MSP2 and MSP3 species were only detected in the Sudano-Sahelien zone, while MSP1 was detected in both Sudano-Sahelian and Sahelian zones, and MSP4 was only found in the Sahelian zone. However the species MSP2, MSP3 and MSP4 contain very few strains and thus we cannot conclude about their geographical pattern (though we can conclude that these species are quite rare compared to MSP1 and MP). For MP and MSP1 the geographical pattern seems to be clear and thus related to the eco-climatic zones (rainfall) rather than the salinity of the soils.

## Discussion

### *Mesorhizobium* strains of *Acacia seyal* and *A. senegal* belong mainly to *M. plurifarium* but also to at least three new species

The MLSA phylogeny based on 5 housekeeping genes showed a higher species diversity of *Mesorhizobium* strains nodulating *A. seyal* and *A. senegal* than previously expected. Most of the strains (60%) clustered with the *M. plurifarium* type strain ORS 1032^T^, as previously reported for some of them [22–25,27]. The rest of the strains were distributed in four new clades, which happen to be new species for at least 3 of them (MSP1 to MSP3) according to the average nucleotide identities of genomes of representative strains in each clade. Unfortunately we did not sequence a representative strain of MSP4 as we did not initially expect this clade in the first results of the single markers phylogenies. The ANI has been proposed as an alternative to DNA-DNA hybridizations (DDH) to infer bacterial species affiliation ([41,42]. If we apply the cut-offs of species delineation as previously published (ANI>95% on 69% of conserved DNA), then the MLSA phylogeny of *Acacia* mesorhizobia fits perfectly with the ANI-based species affiliation. The use of ANI seems to be a good species assessment in the *Mesorhizobium* genus, as there was also no conflict of ANI values between known *Mesorhizobium* species (Table 4, all known species comparison gave ANI <95%). The ANI between reference strains also confirms the belonging of the strain MAFF303099 to a separate species from *M. loti* (89.35% ANI with *M. loti* USDA3471^T^) as suggested by Turner et al. [65] and Wang et al. [66]. Our study also confirms the good performance of the 5 markers used (*atp*D, *dna*J, *gyr*B, *rec*A and *gln*A) in *Mesorhizobium* diversity studies as previously reported for rhizobia [28,35–37,39,53,67].

Our results thus suggest three new species of *Mesorhizobium* nodulating both *Acacia* species. Two of the new clades were anticipated to be separate species from MP as they clustered separately in the IGS spacer phylogeny (as ORS3359 and ORS3324) of the Diouf et al. [22] study, though not in their 16S rDNA phylogeny (this study, [22], [24]). It is interesting to note that MSP1 was only detected in *A. senegal* nodules, while MP was found in the nodules of both *Acacia* species. A recent analysis of nodulation genes clustered *Mesorhizobium* strains (MP, MSP2, MSP3) according to their host of origin: strains from the MSP1 clade (all from *A. senegal*) were clustered with *M. plurifarium* strains from *A. senegal* in their *nod*A, *nod*C and *nif*H phylogenies (Bakhoum *et al*., Microbial Ecology, in press). In the case of *A. seyal* strains, these are all grouped together in the *nod*A and *nod*C phylogenies [33]. The only exception of this latter study was the *nod*C of ORS3324 that grouped with the *nod*C of the *Ensifer arboris* type strain. As we sequenced the full genome of ORS3324, we analyzed its *nod*C in the genome data and found out that it grouped together with the other *A. seyal* mesorhizobia (ORS3359 and ORS3324 sharing 100% nucleotide on *nod*A and *nod*C), thus the *nod*C fragment published in Diouf *et al*. [33] shall be considered as an error.

The nodulation ability of strains was assessed on *A. seyal* and *A. senegal* and all strains were able to nodulate both species, whatever their species affiliation or their *nod*A allele. Such ability is correlated with previous articles showing that strains of the large *M. plurifarium* clade (also described as Cluster U in previous studies) share similar nodulation host range on *Acacia*, *Prosopis* and *Leucaena* species [25,68], and thus their host range shall not explain their geographical distribution.

### The tolerance to NaCl is highly variable among *M. plurifarium* and MSP1 species—whatever their geographical and pedoclimatic origin

A great variability of response to salt of *Acacia* mesorhizobia from MP and MSP1 was found. Conversely, strains of MSP2 to MSP4 did not exhibit high salt tolerance, but given the low number of strains in each of these species it is difficult to conclude to a species effect. Taken altogether, the mesorhizobia strains were mostly salt tolerant at 200 mM (i.e. with a growth at least of 50% compared to the control without salt), as 95% and 43% of strains could still grow at 200 mM and 300 mM of NaCl, respectively. For the species MP, 100% of strains could grow at 200 mM, while 48% and 14% of strains tolerate 300 mM and 400 mM of NaCl, respectively, and one (STM8773) tolerate 500 mM. It has been previously shown that the *M. plurifarium* type strain ORS1032^T^ was more tolerant to heat and salt than several other type strains of *Mesorhizobium* species [31]. In our study, ORS1032^T^ tolerate 400 mM of salt, together with others strains. The MP species could thus be more tolerant than others *Mesorhizobium* species, but a larger study including many strains from the different species is required to infer this question. Another species detected in this study, MSP1, seems well adapted to salt tolerance. The ability of some symbiotically effective strains to tolerate high salinity is promising with regard to improving host plant reestablishment in salt affected soils [46,69].

We investigated possible links between the genetic diversity of mesorhizobia, their salt tolerance and the soils samples characteristics. However, we found a total lack of correlation between these parameters. The most salt-tolerant strains (up to 500 mM, as STM8773 and STM8789) were isolated from non-saline areas. These results are similar to previous studies of Thrall et al. [46] who found also salt-tolerant strains (up to 400 mM) from nodules of *Acacia decurrens* in non-saline sites. Several studies have underlined the lack of correlation between the sampling sites characteristics, the genetic diversity of rhizobia and the tolerance to salinity of rhizobial isolates ([33,45,46]. In Senegal, Diouf et al [22] found a weak influence of soil characteristics (pH and salt) on the distribution of rhizobial populations of *A. seyal* in the Groundnut Basin, as observed in our study at a larger scale. A possible explanation for this lack of correlation could be that the soil is not homogeneous as shown by van Asten et al. [70] who found large differences in salinity and alkalinity levels at short distances in salt affected areas. Such conditions would allow the persistence of various phenotypic traits among rhizobial populations. Rhizobia might also be protected in the nodules, explaining why non-tolerant strains can be detected in highly saline soils. The accumulation of certain compounds, including osmoprotectants (as trehalose and Poly β-hydroxybutyrate) and compatible solutes, may also increase the osmotic tolerance of rhizobia [71–73].

On the other hand, we found a putative geographical pattern of *A. senegal* symbionts between the dryland north part and the center of Senegal. The MSP1 strains were found both in the center and north part of the country while MSP4 and *M. plurifarium* species were found only in the north and the center part of the country, respectively. Such specific distribution of *Mesorhizobium* species has also been observed on *Caragana* spp. symbionts in three eco-regions of China [74]. The geographical pattern of symbionts observed in our study could be linked to the annual rainfall characteristics of each site, as observed for *Vigna unguiculata* symbionts in Senegal [75]. Indeed, the *M. plurifarium* strains were only found in the center of the Sudano-Sahelian zone where annual rainfall is between 500 and 900 mm, while no strain of this clade was found in the Kamb and Dahra sites, located in the arid regions of the sahelian zone where annual rainfall varied between 250 and 500 mm (Fig. 1). This result implies that *M. plurifarium* strains would not be well adapted to dryland conditions. On the other hand, the MSP1 strains from the Kamb soil were shown to be tolerant to water stress when testing their growth with different concentrations of polyethylene glycol [24]. Wade et al [75] found also an eco-geographical diversity of cowpea bradyrhizobia in Senegal marked by the dominance of two genetic types depending on annual rainfall. These authors concluded a possible role of the water regime and the pH in shaping the cowpea bradyrhizobia genotypic distribution, noticing that strains isolated from the northern region were, generally, more adapted to water stress and slightly alkaline soils. The observed geographical distribution of *Acacia* mesorhizobia could thus reflect particular adaptations of each species to specific local conditions as the water regime, but the presence of salt does not seem to be an important structuring factor of *Mesorhizobium* species.

## Supporting Information

S1 Table Genbank accession numbers of all sequences from this study. *Acacia* mesorhizobia from Senegal and type strain of rhizobial species with corresponding GenBank records (accesssion numbers or Gene ID).
(DOCX)

S1 Fig Radar of *Mesorhizobium* strain optical density when grown in TY broth medium with different concentrations of NaCl (100 to 500 mM). The data presented here are at 24h, 41h and 68h post-inoculation with 0.05 OD as a starting point. Each color indicates a concentration of NaCl.
(DOCX)

S2 Fig Histogram of growth for each strain when grown in TY broth medium with different concentrations of NaCl (100 to 500 mM), at 41h post-inoculation. A) Optical density, B) % of growth compared to the same strain growth in TY without salt (B). Standard deviations were calculated from triplicates wells. The arrows indicate the names of the best tolerant strains of *Mesorhizobium* in our bacterial growing conditions.
(DOCX)

S3 Fig Multivariate correspondence analysis of strains, geographical and soil factors. A) Multivariate correspondence analysis factor map for *Mesorhizobium* strains (in blue) with factors: strain tolerance (T100 to T500 corresponding to tolerance to 100 to 500 mM of NaCl, in green), pH of soil of origin (in medium blue, defined into 5 classes: 4, 5, 6, 6.5, 7), species assignment (in black, MP for *M. plurifarium*, MSP1 to MSP4 as defined in the study), name of site of origin (in red), presence of salt in soil of origin (in pale blue, S = Salted (Electroconductivity>4000 μS.cm), MS = moderately salted (400<EC<4000), NS = not salted (EC<400)), plant host (in orange), Ecogeographic zones (in brown), and climatic zone (in pink with Sah for Sahelian and Sud for sudano-sahelian zone). The MCA analysis was performed with MineFactorR under R software. B) Distribution of each factor in the MCA analysis. For Species: MP means *M. plurifarium*, MSP1 to MSP4 correspond to the new species. For NaCl tolerance: the number indicates the concentration in mM in the medium at which the bacteria could still grow >50% compared to the negative control without salt. For soil pH: exact values were transformed into classes by agglomerating close values to the value of 4, 5, 6, 6.5, and 7. For soil salt content, NS means not salted (Electroconductivity <400 uS.cm), MS moderately salted (400<EC<4000 uS.cm), and S salted (EC >4000 uS.cm, as defined by F.A.O.) For Clim (climatic zone): Sah = sahelian zone as defined in the map in Fig. 1. For Ecogeo (Ecogeographical region). BassinArachidier means groundnut basin. For Geo: Geographical origin, indicate the locality of origin of the strains.
(DOCX)
