# Supplementary material for: Genetic and Genomic Diversity Studies of Acacia Symbionts in Senegal Reveal New Species of Mesorhizobium with a Putative Geographical Pattern
Source: PLoS One. 2015 Feb 6;10(2):e0117667. doi: 10.1371/journal.pone.0117667 (PMC4319832; doi:10.1371/journal.pone.0117667)
Supplement: S1 Table — Acacia mesorhizobia from Senegal and type strain of rhizobial species with corresponding GenBank records (accesssion numbers or Gene ID). (DOCX) [file pone.0117667.s001.docx]

S1 Table: *Acacia* mesorhizobia from Senegal and type strains of rhizobial species with corresponding GenBank records (accession numbers or Gene ID).

|  | | | | | | | |
| --- | --- | --- | --- | --- | --- | --- | --- |
| Species/strain | 16S rRNA gene | *recA* | *gyrB* | *atpD* | | *dnaJ* | *glnA* |
| *Mesorhizobium plurifarium* | |  |  |  | |  |  |
| ORS1032^T*^ | Y14158 | AY494824 | AM076347 | AY785350 | | EF504300 | DQ659490 |
| ORS3302 | **KJ609577** | **KJ609604** | **KJ648271** | **KJ648182** | | **KJ648228** | **KJ883336** |
| ORS3356^*^ | DQ859036 | **Genome** | **Genome** | **Genome** | | **Genome** | **Genome** |
| ORS3357 | DQ859037 | **KJ609606** | **KJ648273** | **KJ648184** | | **KJ648232** | **KJ883315** |
| ORS3365^*^ | DQ859039 | **Genome** | **Genome** | **Genome** | | **Genome** | **Genome** |
| ORS3369 | DQ859040 | **KJ609608** | **KJ648275** | **KJ648186** | | **KJ648234** | **KJ883337** |
| ORS3397 | DQ859041 | **KJ609609** | **KJ648276** | **KJ648187** | | **KJ648235** | **KJ883317** |
| ORS3399 | **KJ609578** | **KJ609610** | **KJ648277** | **KJ648188** | | **KJ648236** | **KJ883318** |
| ORS3400 | **KJ609579** | **KJ609611** | **KJ648278** | **KJ648189** | | **KJ648237** | **KJ883319** |
| ORS3404 | DQ859045 | **KJ609612** | **KJ648279** | **KJ648190** | | **ND** | **RDP** |
| ORS3588 | JQ039735 | **KJ609613** | **KJ648280** | **KJ648191** | | **ND** | **KJ883321** |
| ORS3593 | JQ039736 | **KJ609614** | **KJ648281** | **KJ648192** | | **KJ648230** | **ND** |
| ORS3596 | JQ039734 | **KJ609615** | **KJ648282** | **KJ648193** | | **KJ648238** | **KJ883322** |
| ORS3598 | JQ039733 | **KJ609616** | **KJ648283** | **KJ648194** | | **KJ648239** | **KJ883323** |
| ORS3600 | JQ039741 | **KJ609617** | **KJ648284** | **KJ648195** | | **KJ648240** | **KJ883324** |
| ORS3610 | JQ039732 | **KJ609618** | **ND** | **KJ648196** | | **KJ648241** | **RDP** |
| STM8760 | **KJ609580** | **KJ609619** | **KJ648285** | **KJ648197** | | **KJ648242** | **KJ883326** |
| STM8770 | **KJ609582** | **KJ609621** | **KJ648287** | **KJ648199** | | **KJ648244** | **KJ883328** |
| STM8771 | **KJ609583** | **KJ609622** | **KJ648288** | **KJ648200** | | **KJ648245** | **KJ883338** |
| STM8773 | **Genome** | **Genome** | **Genome** | **Genome** | | **Genome** | **Genome** |
| STM8775 | **KJ609585** | **KJ609624** | **KJ648290** | **KJ648202** | | **KJ648247** | **KJ883330** |
| STM8777 | **KJ609586** | **KJ609625** | **KJ648291** | **KJ648203** | | **KJ648248** | **KJ883331** |
| STM8791 | **KJ609587** | **KJ609626** | **KJ648292** | **KJ648204** | | **KJ648249** | **KJ883339** |
| STM8797 | **KJ609588** | **KJ609627** | **KJ648293** | **KJ648205** | | **ND** | **KJ883332** |
| STM8799 | **KJ609589** | **KJ609628** | **KJ648294** | **KJ648206** | | **KJ648250** | **KJ883333** |
| STM8805 | **KJ609590** | **KJ609629** | **KJ648295** | **KJ648207** | | **KJ648251** | **KJ883334** |
| STM8813 | **KJ609591** | **KJ609630** | **KJ648296** | **KJ648208** | | **KJ648252** | **KJ883335** |
| STM8818 | **KJ609592** | **KJ609631** | **KJ648297** | **KJ648209** | | **KJ648253** | **KJ883340** |
| *Mesorhizobium sp.* MSP1 | |  |  |  | |  |  |
| ORS3416 | EU584256 | **KJ609632** | **KJ648298** | **KJ648210** | | **KJ648254** | **KJ883298** |
| ORS3423 | **KJ609593** | **KJ609633** | **KJ648299** | **KJ648211** | | **KJ648255** | **KJ883299** |
| ORS3437 | **KJ609594** | **KJ609634** | **KJ648300** | **KJ648212** | | **KJ648256** | **KJ883300** |
| ORS3443 | **KJ609595** | **KJ609635** | **KJ648301** | **KJ648213** | | **KJ648257** | **KJ883301** |
| ORS3447 | **KJ609596** | **KJ609636** | **KJ648302** | **KJ648214** | | **KJ648258** | **KJ883302** |
| ORS3448 | **KJ609597** | **KJ609637** | **KJ648303** | **KJ648215** | | **KJ648229** | **KJ883303** |
| ORS3450 | **KJ609598** | **KJ609638** | **KJ648304** | **KJ648216** | | **KJ648259** | **ND** |
| ORS3452 | **KJ609599** | **KJ609639** | **KJ648305** | **KJ648217** | | **KJ648260** | **KJ883304** |
| ORS3573 | JQ039728 | **KJ609640** | **KJ648306** | **KJ648218** | | **KJ648261** | **KJ883305** |
| ORS3578 | JQ039731 | **KJ609641** | **KJ648307** | **KJ648219** | | **KJ648262** | **KJ883306** |
| STM8768 | **KJ609600** | **KJ609642** | **KJ648308** | **KJ648220** | | **KJ648263** | **KJ883307** |
| STM8782 | **KJ609601** | **KJ609643** | **KJ648309** | **KJ648221** | | **KJ648264** | **KJ883308** |
| STM8789^*^ | **Genome** | **Genome** | **Genome** | **Genome** | | **Genome** | **Genome** |
| **STM8792** | **KJ609603** | **KJ609645** | **KJ648311** | **KJ648223** | | **KJ648266** | **KJ883310** |
| *Mesorhizobium sp.* MSP2 | |  |  |  | |  |  |
| ORS3359^*^ | DQ859038 | **Genome** | **Genome** | **Genome** | | **Genome** | **Genome** |
| *Mesorhizobium sp.* MSP3 | |  |  |  | |  |  |
| ORS3324^*^ | DQ859034 | **Genome** | **Genome** | **Genome** | | **Genome** | **Genome** |
| *Mesorhizobium sp.* MSP4 | |  |  |  | |  |  |
| ORS3428 | EU584255 | **KJ609648** | **KJ648314** | **KJ648226** | | **KJ648269** | **ND** |
| ORS3628 | JQ039740 | **KJ609649** | **KJ648315** | **KJ648227** | | **KJ648270** | **KJ883313** |
| *Mesorhizobium albiziae* | |  |  |  | |  |  |
| CCBAU61158^T^ | DQ100066 | DQ311090 | JQ013907 | DQ311091 | | JQ013934 | EU249396 |
| *M. alhagi* |  |  |  |  | |  |  |
| CCNWXJ12-2^T^ | EU169578 |  | EU722445 | FJ481887 | |  | FJ481878 |
| *M. amorphae* |  |  |  |  | |  |  |
| ACCC19665^T^ | AF041442 | AY493453 | EF504296 | DQ659488 | | AM076341 | AY494816 |
| *M. australicum* |  |  |  |  | |  |  |
| WSM2073^T^ | AY601516 |  |  |  | |  |  |
| *M. camelthorni* |  |  |  |  | |  |  |
| CCNWXJ40-4^T^ | EU169581 |  |  | GU220802 | |  | GU220798 |
| *M. caraganae* |  |  |  |  | |  |  |
| CCBAU11299 ^T^ | EF149003 | EU249379 | JQ013908 | JQ013925 | | GQ847929 | EU249394 |
| *M. chacoense* |  |  |  |  | |  |  |
| LMG19008^T^ | AJ278249 | AY493460 | EU273806 | DQ659489 | | AM076343 | AY494825 |
| *M. ciceri* |  |  |  |  | |  |  |
| UPM-Ca7^T^ | DQ444456 | AJ294395 | EF504297 | AF169565 | | AM076342 | AJ294367 |
| *M. gobiense* |  |  |  |  | |  |  |
| CCBAU83330 | EF035064 | EF549409 | JQ013909 | JQ013926 | | GQ847928 | EF549481 |
| *M. huakuii* |  |  |  |  | |  |  |
| CCBAU2609^T^ | D127979 | AJ294394 | EF504298 | AF169562 | | AM076344 | AJ294370 |
| *M. loti* |  |  |  |  | |  |  |
| NZP2213^T^ | X67229 | AJ294393 | EU053202 | JQ013927 | | EU273810 | AJ294371 |
| *M. mediterraneum* | |  |  |  | |  |  |
| UPM-Ca36^T^ | L38825 | AJ294391 | EF504299 | AF169564 | | AM076346 | AJ294369 |
| *M. metallidurans* | |  |  |  | |  |  |
| STM2683 | AM930381 | AM930388 | JQ013910 | JQ013928 | | JQ013935 | AM930382 |
| *M. opportunistum* | |  |  |  | |  |  |
| WSM2075^T^ | 10825488 | 10825022 | 10824549 | 10828433 | | 10824117 | 10828678 |
| *M. robiniae* |  |  |  |  | |  |  |
| CCNWYC115^T^ | EU849582 | GQ856506 |  |  | |  | GQ856501 |
| *M. septentrionale* | |  |  |  | |  |  |
| HAMBI2582^T^ | AF508207 | DQ659498 | EF504301 | DQ659491 | | EU273811 | DQ444304 |
| *M. shangrilense* | |  |  |  | |  |  |
| CCBAU65327^T^ | EU074203 | EU672471 |  |  | |  | EU672501 |
| *M. tarimense* |  |  |  |  | |  |  |
| CCBAU83306^T^ | EF035058 | EF549410 | JQ013911 | JQ013929 | | JQ013936 | EF549482 |
| *M. temperatum* |  |  |  |  | |  |  |
| HAMBI2583^T^ | AF508208 | DQ659499 | EF504302 | DQ659492 | | EU273812 | DQ444305 |
| *M. thiogangeticum* | |  |  |  | |  |  |
| SJT^T^ | AJ864462 |  | JQ013912 |  | |  | AM040610 |
| *M. tianshanense* | |  |  |  | |  |  |
| A-1BS^T^ | AF041447 | AJ294392 | EF504303 | AF169577 | | AM076348 | AJ294368 |
| *Rhizobium etli* |  |  |  |  | |  |  |
| CFN42^T^ | 3893174 | 3894384 | 3890921 | 3891786 | | 3892150 | 3892326 |
| * indicate strains which genome was sequenced in this study. Their sequence data are available in EMBL through their project ID (Table 3). | | | | |  |  |  |
| Accession numbers for sequences genes resulting from this study are shown in bold. | | | | | | |  |
