# Supplementary material for: Genetic and Genomic Diversity Studies of Acacia Symbionts in Senegal Reveal New Species of Mesorhizobium with a Putative Geographical Pattern
Source: PLoS One. 2015 Feb 6;10(2):e0117667. doi: 10.1371/journal.pone.0117667 (PMC4319832; doi:10.1371/journal.pone.0117667)
Supplement: S1 Fig — The data presented here are at 24h, 41h and 68h post-inoculation with 0.05 OD as a starting point. Each color indicates a concentration of NaCl. (DOCX) [file pone.0117667.s002.docx]

S1 Figure
