# Supplementary material for: Genetic and Genomic Diversity Studies of Acacia Symbionts in Senegal Reveal New Species of Mesorhizobium with a Putative Geographical Pattern
Source: PLoS One. 2015 Feb 6;10(2):e0117667. doi: 10.1371/journal.pone.0117667 (PMC4319832; doi:10.1371/journal.pone.0117667)
Supplement: S2 Fig — A) Optical density, B) % of growth compared to the same strain growth in TY without salt (B). Standard deviations were calculated from triplicates wells. The arrows indicate the names of the best tolerant strains of Mesorhizobium in our bacterial growing conditions. (DOCX) [file pone.0117667.s003.docx]

S2 Figure.

A.

B.
