# Supplementary material for: Genetic and Genomic Diversity Studies of Acacia Symbionts in Senegal Reveal New Species of Mesorhizobium with a Putative Geographical Pattern
Source: PLoS One. 2015 Feb 6;10(2):e0117667. doi: 10.1371/journal.pone.0117667 (PMC4319832; doi:10.1371/journal.pone.0117667)
Supplement: S3 Fig — A) Multivariate correspondence analysis factor map for Mesorhizobium strains (in blue) with factors: strain tolerance (T100 to T500 corresponding to tolerance to 100 to 500 mM of NaCl, in green), pH of soil of origin (in medium blue, defined into 5 classes: 4, 5, 6, 6.5, 7), species assignment (in black, MP for M. plurifarium, MSP1 to MSP4 as defined in the study), name of site of origin (in red), presence of salt in soil of origin (in pale blue, S = Salted (Electroconductivity>4000 μS.cm), MS = moderately salted (400<EC<4000), NS = not salted (EC<400)), plant host (in orange), Ecogeographic zones (in brown), and climatic zone (in pink with Sah for Sahelian and Sud for sudano-sahelian zone). The MCA analysis was performed with MineFactorR under R software. B) Distribution of each factor in the MCA analysis. For Species: MP means M. plurifarium, MSP1 to MSP4 correspond to the new species. For NaCl tolerance: the number indicates the concentration in mM in the medium at which the bacteria could still grow >50% compared to the negative control without salt. For soil pH: exact values were transformed into classes by agglomerating close values to the value of 4, 5, 6, 6.5, and 7. For soil salt content, NS means not salted (Electroconductivity <400 uS.cm), MS moderately salted (400<EC<4000 uS.cm), and S salted (EC >4000 uS.cm, as defined by F.A.O.) For Clim (climatic zone): Sah = sahelian zone as defined in the map in Fig. 1. For Ecogeo (Ecogeographical region). BassinArachidier means groundnut basin. For Geo: Geographical origin, indicate the locality of origin of the strains. (DOCX) [file pone.0117667.s004.docx]

S3 Figure.

A.

B.
